# Supplementary material for: Confocal Raman Microscopy with Adaptive Optics
Source: ACS Photonics. 2024 Dec 18;12(1):176–84. doi: 10.1021/acsphotonics.4c01432 (PMC11741161; doi:10.1021/acsphotonics.4c01432)
Supplement: Supplementary file 1 — ph4c01432_si_001.pdf [file ph4c01432_si_001.pdf]

Confocal Raman Microscopy with Adaptive Optics: supplemental document

Juan David Muñoz-Bolaños,† Pouya Rajaeipour,‡ Kai Kummer,¶ Michaela Kress,¶ Çağlar Ataman,§ Monika Ritsch-Marte,† and Alexander Jesacher*,†

†*Institute of Biomedical Physics, Medical University of Innsbruck, Müllerstraße 44, 6020 Innsbruck*

‡*Phaseform GmbH, Georges-Köhler-Allee 302, 79110 Freiburg, Germany*

¶*Institute of Physiology, Medical University of Innsbruck, Schöpfstraße 41, 6020 Innsbruck, Austria*

§*Microsystems for Biomedical Imaging Laboratory, Dept. of Microsystems Engineering, University of Freiburg, Georges-Köhler-Allee 101, 79110 Freiburg, Germany*

E-mail: alexander.jesacher@i-med.ac.at

Calculation of shift-free aberration modes

A potential problem with modal wavefront sensing is that the applied test modes can shift the focal spot and therefore change the exposed sample area during the measurement. This happens whenever the pupil is underfilled. Among the popular Zernike modes, those describing coma, which cause transverse shifts, and spherical aberrations, which can cause significant axial shifts of the focus, are particularly problematic. Figure 1(A) visualizes this problem. The three plots show frontal sections through the nominal focus of the confocal intensity PSF calculated for the

parameters of our system (20x, 0.5NA objective, 25 µm core diameter of the collecting fibre, 532 nm excitation wavelength). The application of coma and spherical aberration, in addition to PSF distortion, leads to noticeable lateral and axial shifts due to the small diameter of our excitation beam, whose waist is only about 30% of the pupil radius.

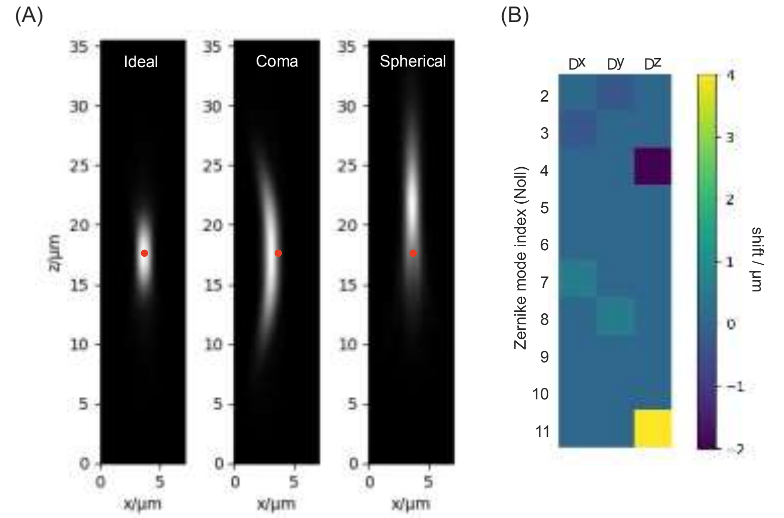

Figure 1: **PSF shifts caused by applied test aberrations.** (A) Left: Frontal cut through the ideal confocal PSF of our system (0.5NA, in air); Middle: Application of primary Zernike coma (1 rad RMS phase) distorts the PSF, but also shifts it along the x-axis; Right: Application of primary spherical aberration leads to a significant axial shift of the PSF. The red points mark the nominal focus point, i.e. the center of the ideal PSF. (B) The colored pixels in the matrix indicate the x-, y- and z-shifts caused by the first ten Zernike modes (leaving out piston) with magnitudes of 1 rad RMS (approx. $0.16\,\lambda$).

A procedure for calculating shift-free modes is outlined in Thayil & Booth.[1] The fist step is to infer the shift vector $\mathbf{s_i} = [\Delta x_i, \Delta y_i, \Delta z_i]$ caused by each mode X_i. Although the authors of [1] followed a measurement approach to find $\mathbf{s_i}$, which also accounts for possible experimental misalignments between the deformable mirror and the objective lens, we can rely on calculations since such misalignments are not possible in our setup (the DPP is always on-axis with the objective due to the mounting thread provided). The calculated shifts for the first ten Zernike modes according to the Noll index scheme build a matrix M, which is shown (in transposed form) in Fig. 1(B). The matrix representation allows us to express the calculation of the total shift vector $\mathbf{s}$ for any given Zernike composition, described by a Zernike magnitude vector $\mathbf{a}$, as follows:

$$\mathbf{s} = \mathrm{M}\mathbf{a} \tag{1}$$

The inversion of the equation using a pseudo-inverse of M allows us to calculate Zernike mode compositions that generate pure spatial shifts of the confocal PSF:

$$\widehat{\boldsymbol{a}} = \mathrm{M}^{\dagger}\mathbf{s} \tag{2}$$

The three columns of $\mathrm{M}^{\dagger}$ contain Zernike magnitude compositions that achieve pure PSF translations along the x-, y- and z-directions. Therefore, pure "shift modes" denoted by D_n (n =1, 2, 3) can be calculated by adding up the Zernike modes with the weights contained in these columns:

$$\mathrm{D_n} \equiv \sum_{i=1}^{N} (\mathrm{M}^{\dagger})_{i,n}\, X_i \tag{3}$$

Figure 2 shows the pure shift modes for our system.

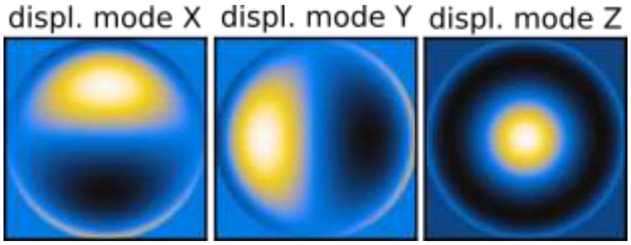

Figure 2: **Pure shift modes for our system parameters**. These modes are removed from the original Zernike set to obtain shift-free modes.

The orthogonal projection technique removes the influence of the pure shift modes from the Zernike basis. These shift modes constitute a 3-dimensional subspace within the 10dimensional space of Zernike modes. The projection of a Zernike mode vector $\mathbf{a}$ onto this subspace can be computed using orthogonal projection:

$$\boldsymbol{a}_{\mathrm{proj}} = \mathrm{M}^{\dagger}\mathrm{M}\,\boldsymbol{a} \tag{4}$$

and shift-free mode vectors $\mathbf{a}_{\mathbf{SF}}$ can be calculated by subtraction:

$$\boldsymbol{a}_{\mathrm{SF}} = \mathrm{M}^{\dagger}\mathrm{M}\,\boldsymbol{a} \tag{5}$$

An example of applying shift-free spherical aberration to the DPP is shown in Fig. 3. An x-z scan of a 5 µm polystyrene bead is taken with the 0.5 NA objective lens. The image is shown in (D). After applying Zernike primary spherical aberration (B) with a magnitude of 0.5 rad, the scan was repeated (E). In addition to image degradation, the bead image shifts along the z-axis by 5 µ. In contrast, applying shift-free spherical aberration (C) leaves the bead image in place (F).

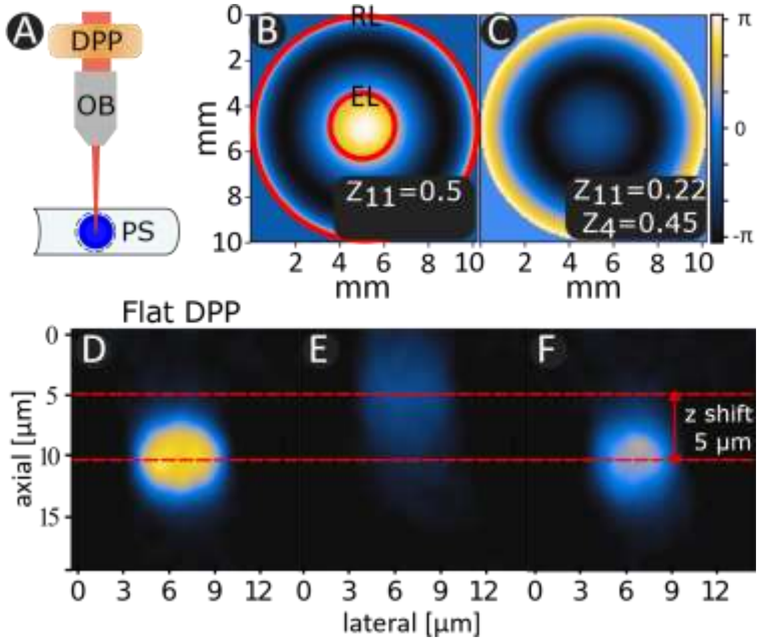

Figure 3: **Application of shift-free spherical aberration**. (A) Experimental setup, showing the DPP, the objective lens and a single PS bead. (B) Zernike primary spherical aberration; the two red rings confine the effective areas traversed by the excitation light (EL) and the Raman back-scattered light (RL). The excitation laser traverses only the central, convex part of the mode and is therefore mainly defocused. (D) x-z-Raman section of the bead, taken with a flat DPP. (E) The image axially shifts by 5 µm when primary spherical aberration is applied to the DPP. (F) No axial shift occurs when the DPP is forming shift-free spherical aberration.

DPP luminescence

We investigated the emission of luminescence from the optofluid inside the DPP. Such emission could potentially deteriorate measurements on weakly Raman active substances. In this measurement, the objective lens and sample were removed and a single Raman spectrum was

recorded with the 532 nm excitation laser and an integration time of five minutes. A second background spectrum was recorded with the DPP removed. The 25µm collection fiber was used. The difference of both spectra is shown in Fig. 4, showing a luminescence contribution of up to 600 counts. Since this background can be subtracted from Raman measurements, it is only its Poisson noise that acts as deteriorating, adding noise of up to about 25 counts.

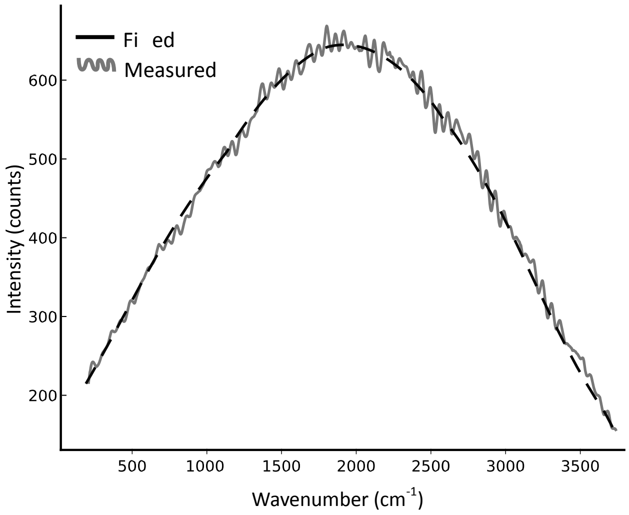

Figure 4: **Luminescence spectrum of the DPP**. The spectrum was acquired in a dark measurement with an integration time of five minutes.

Compensation of an RI mismatch

We investigated the compensation of the RI mismatch introduced by a 1 mm glass slide more thoroughly in a Michelson interferometer. The phase of the light passing the DPP pupil was measured using off-axis interferometry. One arm of the interferometer contained the DPP, the objective lens (0.5NA, 9 mm pupil diameter), and a mirror placed in the focal plane of the objective. A 1 mm glass slab was placed on the mirror to introduce spherical aberrations. The other arm of the interferometer contained a reference mirror.

Figure 5(A) shows the phase profile when the DPP is set to “flat”. The Zernike mode decomposition is shown below. The phase aberration induced by the glass slide is dominated by

defocus (mode 4) and primary spherical aberration with a magnitude of 0.44 µm. Since light traverses the glass twice (mirror double pass), the aberration introduced by the glass slide is 0.22 µm. This corresponds to an effective glass thickness of about 550 µm, while 830 µm are expected considering the precorrection of the objective lens. We hence assume that the objective lens compensates for more than 170 µm glass. Figure 5(B) shows the phase when the DPP was used to compensate for the aberration. While the central part of the pupil appears flat, a residual phase slope remains uncorrected at the pupil rim.

By including this residual phase error in the simulation of the axial Raman response curve, we can model the experimental observation more closely. Figure 5(C) shows the response curves for the aberration-free case (blue), the aberrated case (orange), the ideal DPP correction (green) and the DPP correction under consideration of the residual errors shown in (B) (red). The latter curve has an FWHM value of 13.0 µm, which roughly matches the experimental observation of 14.6 µm. Also, the peak response is close to 60% of the ideal response, which closely matches the experimental result.

References

(1) Thayil, A.; Booth, M. Self-calibration of sensorless adaptive optics microscopes. *Journal of the European Optical Society* 2011, *6*, 11045–8.

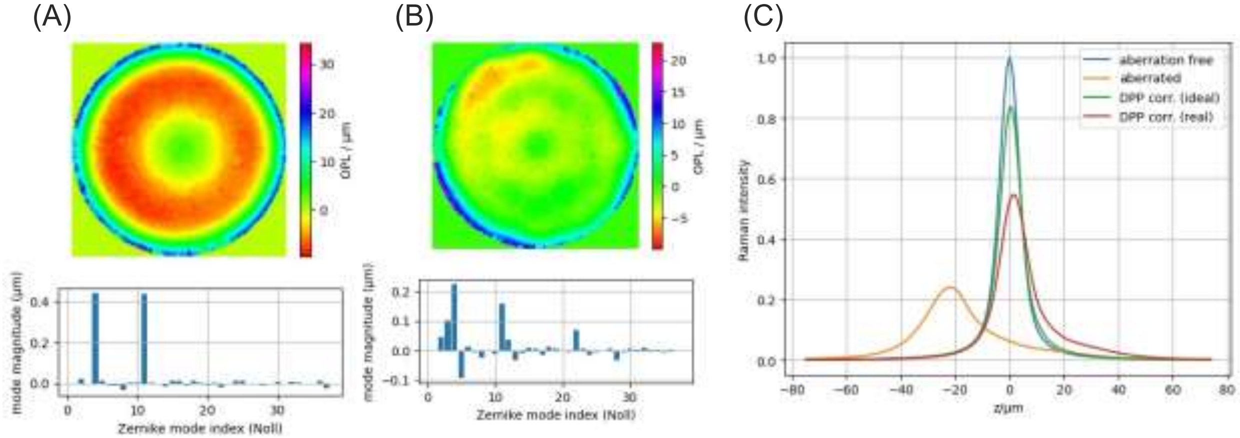

Figure 5: **Interferometric measurement of DPP phase for the compensation of an RI mismatch**. (A) Phase of light in the DPP pupil, after having traversed the 1 mm glass slab twice. (B) Phase after DPP correction. (C) Axial Raman response curves for the aberration-free (blue), aberrated (orange), ideal corrected (green) and real corrected (red) cases.
